# Does stopping at C7 in long posterior cervical fusion accelerate the symptomatic breakdown of cervicothoracic junction?

**DOI:** 10.1371/journal.pone.0217792

**Published:** 2019-05-31

**Authors:** Dong-Ho Lee, Jae Hwan Cho, Jin Il Jung, Jong-Min Baik, Deuk Soo Jun, Chang Ju Hwang, Choon Sung Lee

**Affiliations:** 1 Department of Orthopedic Surgery, Asan Medical Center College of Medicine, University of Ulsan, Seoul, Republic of Korea; 2 Department of Orthopedic Surgery, Gil Medical Center College of Medicine, University of Gachon, Incheon, Republic of Korea; George Washington University, UNITED STATES

## Abstract

**Object:**

To compare the clinical and radiological outcomes between patients with long posterior cervical fusion (PCF) in which fusion stopped at C7 versus patients in which fusion crossed the cervicothoracic junction (CTJ).

**Methods:**

The patients were divided into 2 groups on the basis of the lower-most instrumented vertebra (LIV); C7 group patients (n = 25) and upper thoracic (UT) group (n = 21). We analyzed the visual analogue scale of arm/neck pain, Japanese Orthopedic Association (JOA) score, and neck disability index (NDI). And we also measured the following parameters: (1) pseudomotion of fused segments; (2) C2–C7 sagittal vertical axis; (3) T1 slope; and (4) C2–C7 lordosis.

**Results:**

Arm and neck pain were similar in both groups pre- and postoperatively. Interestingly, mean postoperative NDI score in the UT group was significant worse when compared with the C7 group (9.7±4.6 vs. 14.2±3.7, p = 0.006). Although UT patients had longer fusion levels, the fusion rates were not significantly different between the C7 and UT groups (96.0% vs. 90.5%; p = 0.577). The radiographic parameters did not show any significant differences between the groups at final follow-up.

**Conclusions:**

Our study demonstrates that multi-level PCF stopping at C7 does not negatively affect C7-T1 segment failure, fusion rate, neck pain, neurologic outcomes, and global sagittal alignment of the cervical spine. Hence, it is unnecessary to extend the long PCF levels caudally across the healthy CTJ for fear of development of adjacent segmental disease (ASD) at the C7-T1 segment.

## Introduction

Long posterior cervical fusion surgery (PCF) is often performed for multi-level radiculopathy, myelopathy, or severe kyphotic deformity. And the increase in PCF procedures is likely related to an aging patient population, who are more likely to present with a greater severity of stenosis at multiple levels necessitating a posterior approach [1,2]. Also, incidence of posterior cervical fusions performed in the US has increased in all patients and in those with rheumatoid arthritis [3]. Consistent with other junctional regions of the spine, the cervicothoracic junction (CTJ) has significant morphological variations due to the transition from the fairly mobile, lordotic cervical spine to the more rigid, kyphotic thoracic spine [4–8]. As a result, the CTJ experiences significant static and dynamic stress [8]. Since the cervicothoracic junction (CTJ) represents a unique region that shifts from the mobile lordotic cervical spine to the rigid kyphotic thoracic spine, stopping long fusion at C7 may accelerate adjacent segmental disease (ASD), thus requiring revision surgeries at the C7-T1 segment. While surgeons commonly recommend extending cervical fusion into the thoracic spine to protect the adjacent levels, we did not find any direct evidence to support this procedure. Therefore, the purpose of this study is to compare the clinical and radiological outcomes between patients with long PCF in which fusion stopped at C7 versus patients in which fusion crossed the CTJ.

## Materials and methods

This study was an observational, retrospective cohort study approved by the Institutional Review Board of the Asan Medical Center, Ulsan university school of medicine, AMCIRB (protocol number 2018–0637). All data were fully anonymized before we accessed them and our IRB required informed consent. The patients were verbally informed about the objectives and were included only after reading and signing the written informed consent statement.

### Study population

After institutional review board approval was obtained, we conducted a retrospective review of all patients who underwent minimum 3-level posterior cervical fusion surgery by a single surgeon (DHL). The inclusion criteria were as follows: 1) patients with cervical spondylotic myelopathy or multi-level myeloradiculopathy; 2) those who underwent minimum 3-level posterior cervical fusion surgery; and 3) a follow-up period ≥24 months. Patients with additional posterior surgery procedures, infection, or revision surgeries were excluded, along with patients with a follow-up of <24 months. We reviewed the clinical records and radiographic data of 54 consecutive PCF alone operated cases for cervical spondylotic myelopathy, or myeloradiculopathy between January 2010 and December 2016 were screened for eligibility. Among them, eight of the 54 patients were excluded due to follow-up loss and we included 46 patients (27 men, 19 women; mean age, 63.1±10.4 years; follow-up, 38.4±18.5 months) with minimum 3-level PCF and at least a 2 year follow-up period. The patients were divided into 2 groups on the basis of the lower-most instrumented vertebra (LIV). C7 group patients (n = 25) underwent a long fusion stopping at C7. In upper thoracic (UT) group (n = 21), LIVs were T1 (n = 13), T2 (n = 6), or T3 (n = 2). The decision making that led to stopping thoracic level determines the decompression level according to the location of pathologic lesion and the lower-most instrumented vertebra was determined to resolve this instability of decompressed levels.

### Surgical procedure and postoperative management

All patients underwent minimum 3-level posterior cervical fusion surgery using midline approach, and received additional foraminotomy, if the patients had stenotic neural foramens on preoperative magnetic resonance imaging as well as clinically concordant arm pain. The patient is placed prone on a four-post frame using a horseshoe-type headrest or three-point pin fixation device. The shoulders are pulled caudally by a heavy bandage for intraoperative lateral fluoroscopic imaging of the lower cervical spine. A skin incision is made to the appropriate level for instrumentation. The paravertebral muscles are dissected laterally to expose the lateral margins of the articular masses completely for exact determination of the screw insertion point. First, the spinous process was excised, and the screw insertion point was marked and laminectomy was performed through a microscope. After screw (Synapse, DePuy Synthes, CA, US) insertion, posterolateral fusion was performed with a local bone using the resected spinous process and lamina. In these all cases, a lateral mass screw was used for C3 to C6 and a pedicle screw was inserted for the lower part through C-arm guided. The postoperative neck collar period was 12 weeks in all patients.

### Demographic and outcome analysis

Demographic data, including age, sex, operative level, and follow-up period were retrospectively collected from the electronic medical records. Clinical data were prospectively collected by 1 clinical researcher during the preoperative period and during each patient visit. Neck and arm pain were assessed according to the visual analogue scale (VAS), Japanese Orthopedic Association (JOA) score, and the neck disability index (NDI) were also estimated. And to evaluate fusion status and sagittal alignment, we also measured the following parameters through the plain cervical spine (standing position) X-ray preoperatively, and at the last follow-up: (1) pseudomotion of fused segments; (2) C2–C7 sagittal vertical axis (SVA); (3) T1 slope; and (4) C2–C7 lordosis. The C2-C7 SVA was obtained through measuring the distance from the posterior-superior corner of C7 to a vertical line that bisected the C2 centroid. The T1 slope is the angle created from a line tangential to the superior end plate of T1 and a horizontal line. Lastly, the C2-C7 lordosis was measured using the Cobb angle between the inferior end plate of C2 to the inferior end plate of C7.

### Assessment of fused segments

An independent reviewer assessed radiographic fusion following PCF in a blind manner. A diagnosis of pseudarthrosis was made by observing any one of the following: (1) remaining interspinous distance (ISD) change >1 mm on flexion/extension lateral X-ray magnified by ≥150% [9]; (2) grafted local bone is not confirmed to be fused with cortical-host bone (lateral mass area) or non-bony bridge between grafted local bone and host bone on computed tomography (CT) scans. All of the patients were instructed to maximally flex the neck (chin to chest) and then extend the neck (face toward ceiling) when obtaining the radiographs, and the distance between the tube and target was estimated at approximately 72 inches. The most identifiable landmark around the tip of the spinous process at each level was chosen; this landmark was identifiable on both flexion and extension lateral radiographs simultaneously on the same monitor. All the CT scans were obtained at 0.75-mm intervals with coronal and sagittal reconstruction. The presence of pseudarthrosis was evaluated at final follow-up. Secondary confirmation was performed with radiologist's readings and the same results were confirmed.

### Statistical analysis

Data management and statistical analyses were performed using the Statistical Package for the Social Sciences (SPSS, version 18.0; SPSS Inc., Chicago, IL). The distributions of variables are presented as means and standard deviation. Patients were assigned to 2 groups: a C7 group and UT group. The between-group differences in demographic data and fusion rate at final follow-up were analyzed using the Mann Whitney U-test and Pearson chi-square test. To compare the clinical results and radiologic parameters, between theses 2 groups was analyzed using the Student t test. The pre- and postoperative clinical and radiological data were compared using the Mann Whitney U-test and Wilcoxon signed rank test. A p value <0.05 was considered statistically significant. Power analysis for a t test was conducted in G-POWER program to determine a sufficient sample size using an alpha of 0.05, power of 0.80, a large effect size (w = 0.85) and 5 degrees of freedom. Based on the aforementioned assumptions, the desired sample size was 36.

## Results

**Fig 1:**
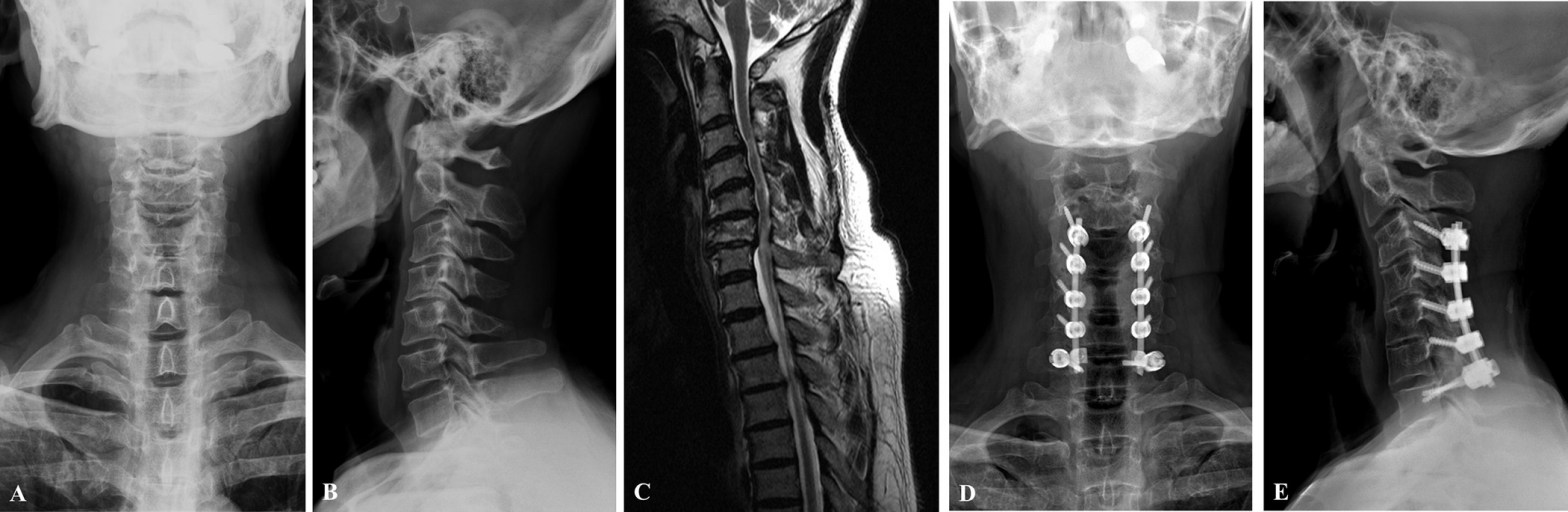
Case C7 group. Case of a 50-year-old male patient who underwent posterior decompression and fusion surgery with posterior instrumentation from C3 to C7 due to cervical myeloradiculopathy. (A), (B) C-spine anteroposterior and lateral X-ray, preoperatively. (C) T2 weighted MR image (sagittal), preoperatively (D), (E) postoperative X-ray at final follow-up.

**Fig 2:**
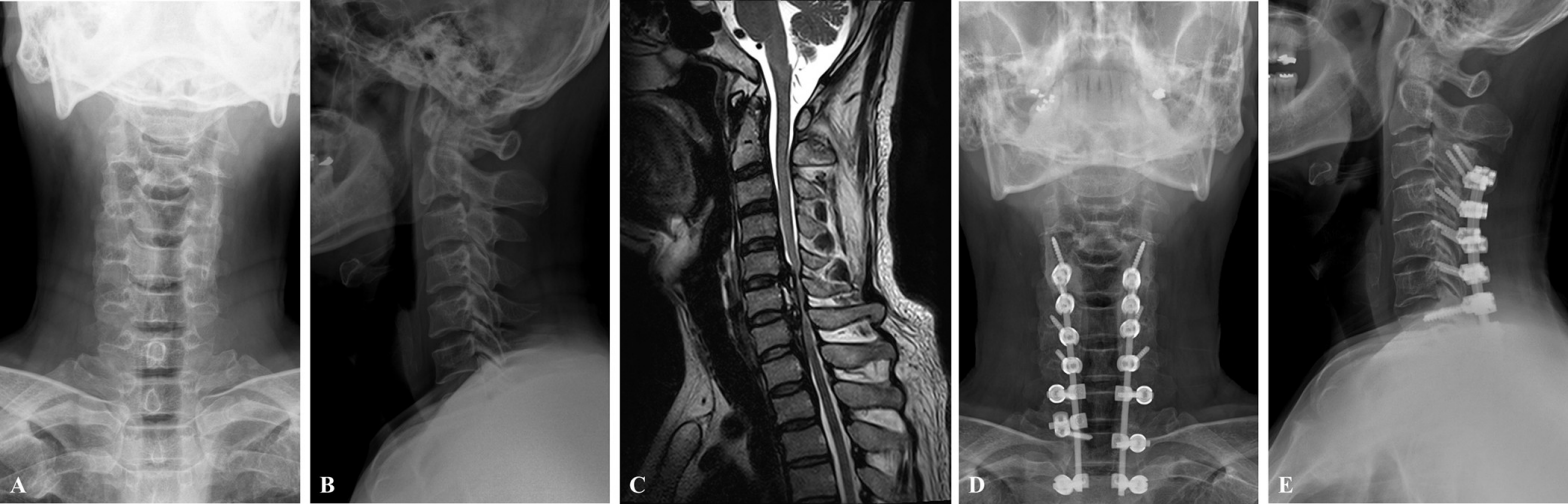
Case UT group. Case of a 56-year-old male patient who underwent posterior decompression and fusion surgery with posterior instrumentation from C3 to T2 due to cervicothoracic myeloradiculopathy. (A), (B) C-spine anteroposterior and lateral X-ray, preoperatively. (C) T2 weighted MR image (sagittal), preoperatively (D), (E) postoperative X-ray at final follow-up.

All surgical procedures were performed between the C2 to T3 levels, and the C7 group contains patients who underwent PCF surgery between C2 and C7 levels. On the other hand, twenty one patients of 46 underwent PCF surgery at least T1 level (UT group); LIVs, T1 (n = 13), T2 (n = 6), or T3 (n = 2). The operation cases for each group are presented at Fig 1 (C7 group) and Fig 2 (UT group).

### Patient demographics

There were no significant differences in age, gender, or follow-up period between the two groups. However, the UT group showed statistically different results at the operation level than the C7 group (5.38 ± 0.59 vs. 7.29 ± 1.72, p <0.001) (Table 1).

**Table 1:** Comparison of study patient demographics.

	C7^¶^ (n = 25)	UT^§^ (n = 21)	P value
Age (years)	-	-	-
Mean ± SD*	60.95 ± 10.91	65.29 ± 9.87	0.181
Sex	-	-	-
Male: Female	15: 10	12: 9	1.000
Follow-up periods (month)	-	-	-
Mean ± SD	38.14 ± 15.22	38.88 ± 22.67	0.426
Operation level	-	-	-
Mean ± SD	5.38 ± 0.59	7.29 ± 1.72	< 0.001
Last instrumented vertebra (LIV)	-	-	-
C7	25	-	-
T1	-	13	-
T2	-	6	-
T3	-	2	-
Fusion rate (final follow-up)	24/25 (96.0%)	19/21 (90.5%)	0.577

*SD, standard deviation

¶C7, fusion stopping at C7 level

§UT, fusion stopping at upper thoracic level

### Fusion rate

Although UT patients had longer fusion levels, the fusion rates were not significantly different between the C7 and UT groups at final follow-up (96.0% vs. 90.5%; p = 0.577) (Table 1).

### Clinical outcomes

Arm and neck pain were similar in both groups pre- and postoperatively, and there were statistically no difference between two groups. Mean JOA score was significantly worse in UT group preoperatively (6.76±2.02 vs. 12.00±1.50, p<0.001), but it improved to a similar degree as the C7 group after surgery (15.43±2.06 vs. 15.18±1.43, p = 0.294). Interestingly, mean postoperative NDI in the UT group was significant worse when compared with the C7 group (9.80±4.55 vs. 14.24±3.70, p = 0.006) (Table 2).

**Table 2:** Changes of clinical outcomes between preoperative periods and final follow-up in both groups.

	C7^‡^ (n = 25)	UT^∮^ (n = 21)	P value
Neck VAS*	(Mean ± SD^†^)	(Mean ± SD^†^)	-
Pre-Op.^∫^	6.24 ± 1.45	6.18 ± 1.78	0.794
Final follow-up	1.38 ± 1.32	2.06 ± 1.48	0.220
Arm VAS*	-	-	-
Pre-Op.^∫^	6.48 ± 1.44	6.65 ± 1.32	0.816
Final follow-up	0.95 ± 1.20	1.53 ± 1.23	0.170
JOA^¶^ score	-	-	-
Pre-Op.^∫^	12.00 ± 1.50	6.76 ± 2.02	< 0.001
Final follow-up	15.43 ± 2.06	15.18 ± 1.43	0.294
NDI^§^	-	-	-
Pre-Op.^∫^	22.19 ± 5.79	22.88 ± 3.18	0.367
Final follow-up	9.80 ± 4.55	14.24 ± 3.70	0.006

*VAS, Visual analogue scale

¶JOA, Japanese orthopedic association

§NDI, Neck disability index

†SD, standard deviation

‡C7, fusion stopping at C7 level

∮UT, fusion stopping at upper thoracic level

∫Op., operation

Analyzing each NDI questionnaire details, there was no difference between two groups in the most questions, but the UT group showed statistically significant worse in question 7, which is the work, preoperatively (p = 0.002). And both groups demonstrated improvement after surgery on all NDI questions. However, UT group showed statistically significant worse results compared to C7 group in question 1 (p = 0.012), 3 (p<0.001), 7 (p<0.001), and 10 (p<0.001) at final follow-up (Table 3).

**Table 3:** Compare the details of NDI questionnaire between C7 and UT group.

NDI*	C7^§^ (n = 25)	UT^†^ (n = 21)	P value
Question 1 (Pain intensity)	(Mean ± SD^¶^)	(Mean ± SD^¶^)	-
Pre-Op.^‡^	2.43 ± 0.75	2.24 ± 0.56	0.542
Final follow-up	0.95 ± 0.67	1.65 ± 0.70	0.012
Question 2 (Personal care)	-	-	-
Pre-Op.^‡^	1.95 ± 0.87	1.71 ± 0.47	0.504
Final follow-up	0.86 ± 0.57	0.71 ± 0.47	0.523
Question 3 (Lifting)	-	-	-
Pre-Op.^‡^	2.95 ± 0.74	3.47 ± 0.71	0.064
Final follow-up	1.29 ± 0.64	2.24 ± 0.56	< 0.001
Question 4 (Reading)	-	-	-
Pre-Op.^‡^	2.05 ± 0.38	1.88 ± 0.33	0.432
Final follow-up	1.00 ± 0.45	0.94 ± 0.24	0.794
Question 5 (Headaches)	-	-	-
Pre-Op.^‡^	1.00 ± 0.84	1.12 ± 0.60	0.561
Final follow-up	0.52 ± 0.51	0.47 ± 0.51	0.794
Question 6 (Concentration)	-	-	-
Pre-Op.^‡^	2.05 ± 0.67	1.65 ± 0.49	0.101
Final follow-up	0.86 ± 0.57	0.76 ± 0.44	0.728
Question 7 (Work)	-	-	-
Pre-Op.^‡^	3.10 ± 0.70	3.94 ± 0.75	0.002
Final follow-up	1.33 ± 0.58	2.88 ± 0.78	< 0.001
Question 8 (Driving)	-	-	-
Pre-Op.^‡^	2.38 ± 0.67	1.94 ± 0.24	0.052
Final follow-up	1.05 ± 0.59	1.12 ± 0.48	0.772
Question 9 (Sleeping)	-	-	-
Pre-Op.^‡^	1.62 ± 0.67	1.76 ± 0.44	0.383
Final follow-up	0.76 ± 0.44	0.88 ± 0.48	0.581
Question 10 (Recreation)	-	-	-
Pre-Op.^‡^	2.67 ± 0.79	3.18 ± 0.64	0.060
Final follow-up	1.19 ± 0.51	2.53 ± 0.94	< 0.001

*NDI, Neck disability index

¶SD, standard deviation

§C7, fusion stopping at C7 level

†UT, fusion stopping at upper thoracic level

‡Op., operation

### Radiologic parameters

No patient in either group had any obvious instability or disc breakdown requiring revision surgeries at caudal adjacent segments. Additionally, the radiographic parameters indicating sagittal alignment including C2–C7 sagittal vertical axis, C2–C7 lordosis and T1 slope did not show statistically any significant differences between the groups before and after surgery (Table 4).

**Table 4:** Changes of radiological parameters between preoperative periods and final follow-up in both groups.

	C7^§^ (n = 25)	UT^†^ (n = 21)	P value
C2-C7 Lordosis (degree)	(Mean ± SD^¶^)	(Mean ± SD^¶^)	-
Pre-Op.^‡^	12.95 ± 6.11	14.82 ± 7.06	0.601
Final follow-up	7.05 ± 6.42	6.12 ± 5.33	0.772
C2-C7 SVA* (mm)	-	-	-
Pre-Op.^‡^	22.95 ± 11.14	21.04 ± 13.57	0.399
Final follow-up	28.98 ± 10.59	33.07 ± 11.91	0.281
T1 Slope (degree)	-	-	-
Pre-Op.^‡^	27.86 ± 7.74	24.18 ± 6.35	0.083
Final follow-up	22.71 ± 9.08	23.59 ± 10.36	0.885

* SVA, Sagittal vertical axis

¶SD, standard deviation

§C7, fusion stopping at C7 level

†UT, fusion stopping at upper thoracic level

‡Op., operation

### Complications

No major neurologic or wound complications were observed except for 1 case, minimal screw pulled-out in C7 group and 2 cases, mild distal junctional kyphosis (DJK) and minimal screw pulled-out at distal level in UT group. However, there was no revision surgery in both groups.

## Discussion

The prevalence of clinical adjacent-segment pathology after cervical spine surgery has been reported to range from 1.6% to 4.2% per year, with reoperation rates for clinical adjacent segment pathology approximately 0.8% per year [10]. And there are still many debates about the cause of ASD after spine surgery. Also, the etiology of radiographic ASD defined as degenerative findings at the adjacent segments found on imaging modalities and clinical ASD, defined as symptoms thought to be related to degenerative changes, remains a debate [11,12]. In vitro biomechanical studies have further evaluated the kinematic challenges that occur at the adjacent levels [13]. In particular, biomechanical studies have shown increased intradiscal pressures in the C7-T1 segment after instrumentation of the lower cervical spine [8]. However, other groups have recently claimed that the adjacent disc degeneration may simply be part of the natural progression of cervical spondylosis [14].

Furthermore, it is very difficult to determine fusion at cervicothoracic junction (CTJ) where the spinal curvature changes and the mobile cervical spine meet the rigid thoracic spine for avoiding ASD. And the anatomical and biomechanical complexities of posterior spine surgery crossing the cervicothoracic junction and other junctional regions pose particular treatment challenges [4,15]. Most of the literature discusses with the possibility of long-term survival through long-level fusion to thoracolumbar and lumbar spine joints, but there is little data on optimal caudal "end level" determinations in posterior cervical fusion surgery. The purpose of this study is to answer the question of whether the long posterior cervical fusion surgery should be routinely performed cross the cervicothoracic junction. So, we have compared the clinical and radiological outcomes between patients with long posterior cervical fusion in which fusion stopped at C7 versus patients in which fusion crossed the cervicothoracic junction.

First of all, C7 and UT groups had similar clinical results. Arm and neck VAS were similar in both groups pre- and postoperatively, and there were statistically no difference between two groups. However, mean JOA score was significantly worse in UT group preoperatively (6.76±2.02 vs. 12.00±1.50, p<0.001), but it improved to a similar degree as the C7 group after surgery (15.43±2.06 vs. 15.18±1.43, p = 0.294). Interestingly, mean postoperative NDI score was improved both groups however, in the UT group, was significant worse when compared with the C7 group (9.80±4.55 vs. 14.24±3.70, p = 0.006). So we analyzed each question details in NDI questionnaire and found that the worse result is seen in the UT group in question 1 corresponding to pain intensity, question 3 (Lifting), question 7 (Work), and question 10 (Recreation). Particularly in question 1 (Pain intensity), NDI questionnaire showed less improvement in UT group comparing with VAS improvement in both groups. We thought that because each NDI question had 5 choices compared with 10 choices in VAS, these differences seemed to be that results. As for the results of the other question 3, 7, and 10, we thought that there were much subjective intervention and individual variation because of relating to the daily life.

Secondly, the radiological parameters and fusion rate did not differ statistically in the two groups. Interestingly, in this present study, although UT patients had longer fusion levels, the fusion rates were not significantly different between the C7 and UT groups at final follow-up (96.0% vs. 90.5%; p = 0.577). And the fusion rate was lower in the UT group rather than C7 group. Schroeder et al. [16] recommended that the multilevel posterior cervical fusions should be extended to T1, as stopping a long construct at C7 increase the rate of revision. Their study divided the patients into the 3 groups (fusion terminating at C7, T1, and T2-T4). The rate of revisions in C7, T1, and T2-T4 were 35.3%, 18.3%, and 40%, respectively (p = 0.008) And multivariate linear regression analysis showed that odds of revision in patients whose construct terminated at C7 were 2.29 times more than T1 group (p = 0.02), but no difference between stopping at T1 and T2-4 was identified. And Truumees at al. [17], in multi-center study with 177 patients, suggested that extension of a posterior cervical fusion into the thoracic spine leads to lower pseudarthrosis rate, whereas stopping C7 level yields lower operation time and estimate blood loss et al., and demonstrated that there are different benefits for each approach. Also, they reported that there were no statistically differences in clinical, radiological outcomes and pseudarthrosis rate between two groups (stopping at C7 vs. stopping at thoracic spine). These results are similar to our clinical and radiological results. Given the limited information available and the conflicting results from these two studies, we suggest that our clinical results had been conducted objectively by a single researcher and the follow-up period for more than two years is sufficient to determine the fusion rate and pseudarthrosis. And we also tried to improve accuracy by using CT as well as X-ray for pseudarthrosis and fusion rate analysis.

Our present study had several limitations of note, including those associated with its retrospective observational design, such as the small number of patients and the single institution assessed. And co-morbidities of the patients (e.g. diabetes, osteoporosis, rheumatic disease, etc.) were not measured in this study. Moreover, although several criteria were used to minimize the false-positive and -negative results for pseudarthrosis diagnosis after PCF surgery, fusion assessment was not performed by more than 2 observers. However, in the present study, we assessed the fusion rate and clinical outcomes after PCF at 2 years postoperatively, which has not been performed previously.

## Conclusions

Our study demonstrates that multi-level PCF stopping at C7 does not negatively affect C7-T1 segment failure, fusion rate, neck pain, neurologic outcomes, and global sagittal alignment of the cervical spine. Of course, the sample size of this study was small and the follow-up period was only two years. So no complications such as ASD requiring treatment were found. However, our study shows that unnecessary long fusion across the cervicothoracic junction is likely to deteriorate postoperative neck function (worse NDI scores). These results suggest that cervical decompression and fusion without extending to the thoracic spine does not increase the rate of early adjacent segment disease or radiographic parameters, and that as demonstrated in the other cohort, surgeries extending into the thoracic spine may result with increased postoperative neck pain and therefore may be advisable to avoid.

## Supporting information

S1 Table Raw data: Patients demographics, clinical and radiologic parameters. Pt., Patients; LIV, Last Instrumented Vertebra; OP, Operation; F/U, Follow-Up; NDI, Neck Disability Index; VAS, Visual Analogue Scale; JOA, Japanese Orthopedic Association; T2-T7, T2-T7 Lordosis (degree); T1S, T1 Slope (degree); SVA, C2-C7 SVA (mm); F, Female; M, Male; O, Fused state; X, Non-fused state.
(XLSX)

S2 Table Raw date: Details of NDI (neck disability index) questionnaire. Pt., Patients; Q, Question; C7, fusion stopping at C7 level; UT, fusion stopping at upper thoracic level.
(XLSX)
